# An Innovative Approach of Using a Bio-Based Polyurethane Elastomer to Overcome the “Magic Triangle” in Tires

**DOI:** 10.3390/ma18030603

**Published:** 2025-01-28

**Authors:** Xin Wang, Dexian Yin, Zhi Chen, Xiuying Zhao, Xin Ye, Shikai Hu

**Affiliations:** 1 State Key Laboratory of Organic-Inorganic Composites, Beijing University of Chemical Technology, Beijing 100029, China; 2 Beijing Engineering Research Center of Advanced Elastomers, Beijing University of Chemical Technology, Beijing 100029, China

**Keywords:** CO_2_-based, bio-based, polyurethane elastomer, magic triangle

## Abstract

Rubber tires are fundamental components of modern society and industrial operations, holding an irreplaceable position in the global manufacturing and transportation sectors. The potential for traditional rubber tires to enhance performance is gradually approaching its limits, rendering it challenging to further improve low rolling resistance, high wet-skid resistance, and high wear resistance (called “magic triangle”). Moreover, the reliance on petroleum resources for rubber hinders the sustainable development of rubber tires. In this work, a series of novel polyurethane (PU) elastomers with potential applications in high-performance automotive tires were synthesized by CO_2_-based poly(propylene carbonate) diol and bio-based poly(propylene oxide) glycol (PO3G). The comprehensive influences of PO3G on the thermal, mechanical, rolling resistance, and wear properties of the elastomers were systematically investigated. The results illustrated that increasing the PO3G content significantly enhanced the wear resistance by 98.43% and the wet-skid resistance by 73.21% and reduced the rolling resistance by 15.38% of the elastomers compared to commercial green tires (HT166). The rational design strategy of PU elastomers not only effectively addresses the “magic triangle” challenge in the tire industry but leverages CO_2_ to contribute to the sustainable development of the automotive sector.

## 1. Introduction

Tires are indispensable component of automobiles and provide vehicles with traction, safety, and comfort, playing a crucial role in the transportation sector [1,2]. The demand for tires is substantial and extensive, projecting to reach approximately 5 billion units by the end of 2030 [3]. High-performance tires are typically made of rubber nanocomposites produced by reinforcing solution-polymerized styrene-butadiene rubber, high-cis-polybutadiene rubber, and natural rubber with carbon black [4,5].

As is well known, wet-skid resistance, rolling resistance, and wear resistance are referred as the “magic triangle” in tire industry [6]. Specifically, enhancing any two of these properties often results in the compromise of the third. Rubber tires often increase rolling resistance while improving wet-skid resistance and wear resistance, making it challenging to perfectly solve the “magic triangle” issue [7], and the potential for rubber tires to balance the “magic triangle” is nearing its limit. Furthermore, rubber tires predominantly depend on non-renewable petroleum resources, hindering the sustainable development of the tire industry. Consequently, investigating innovative tires that are independent of petroleum and capable of effectively balancing the challenge of the “magic triangle” holds significant importance.

Polyurethane (PU), a block copolymer composed of a soft segment (polyol) and a hard segment (isocyanate and chain extender), displays significant design flexibility in structure and adjustable performance characteristics, with wide applications in fields such as aviation, medicine, electronics, and transportation [8,9,10,11,12]. Additionally, PU exhibits high wear resistance and tear resistance [13], indicating its tremendous application potential in tire industry [1]. It is worth noting that the lifecycle of PU-tread rubber is consistent with that of traditional rubber tires, meaning that waste tires can still be recycled and reused. The application of bio-based PU tires can significantly contribute to the bio-based substitution of downstream products made from discarded tires. For instance, collecting the shredded debris from bio-based waste tires can be used to produce bio-based vibration-damping and isolation products for rail transportation [14,15,16,17]. Nevertheless, PU is mostly synthesized from non-renewable petrochemical resources [18,19], the excessive exploitation of which leads to significant greenhouse gas emissions (CO_2_), contributing to global warming and intensifying the energy crisis and environmental pollution [20].

CO_2_ is a plentiful and cost-effective renewable resource that presents opportunities for effective utilization, thereby not only diminishing greenhouse gas emissions but also aligning with carbon neutrality strategies and thereby promoting sustainable development [21]. Poly(propylene carbonate) diol (PPCD) is an aliphatic polycarbonate diol synthesized by copolymerizing CO_2_ and propylene oxide, and the development and application of PPCD can facilitate CO_2_ capture and utilization [22]. Studies have shown that PU synthesized from PPCD exhibits high mechanical strength and good thermal stability, making it valuable in the fields of adhesives, coatings, and foams [23,24,25]. However, the generally high glass transition temperature (*T_g_*) of PPCD gives rise to high hardness, poor flexibility, and difficulties in processing, which is why its application in tread rubber has been rarely explored in previous studies [26,27].

Furthermore, bio-based polyols recently have garnered widespread attention in the PU industry. Poly(propylene oxide) glycol (PO3G) has become one of the most studied bio-based polyols, and it is synthesized by bio-based 1,3-propanediol [28], offering a reduction of 42% in greenhouse gas emissions compared to petrochemical products like poly(tetramethylene-ether) glycol [29]. PO3G exhibits the benefits of renewability and biodegradability, thereby positioning it as a promising alternative to petroleum-based polyols in PU synthesis [30]. Characterized by its odd carbon chain structure [31] and abundant ether bonds, PO3G significantly enhances the molecular chain flexibility [32]. However, PO3G presents poor mechanical properties owing to the low cohesive energy of ether bonds, which limits its application in the tire industry. Therefore, achieving an effective combination of PPCD and PO3G to prepare PU with both high mechanical properties and low *T_g_* could have a profound impact on reducing the use of petroleum-based materials and broadening the application of PU in the tire industry.

In this work, a series of novel bio-based PPCD/PO3G-PU elastomers were synthesized using PO3G and PPCD as the soft segment (SS) and p-phenylene diisocyanate (PPDI) and trimethylolpropane allyl ether (TME) as the hard segment (HS). The comprehensive influences of the PO3G on the thermal properties, mechanical properties, and dynamic mechanical properties as well as the wear resistance and rolling resistance of the elastomers were systematically investigated, with a focus on their potential application in environmentally friendly automobile tires. The PPCD/PO3G-PU elastomers can reduce the reliance on petrochemical resources and effectively balance the “magic triangle”. However, the industrial production of PPCD/PO3G-PU elastomers faces significant challenges due to cost limitations. With advancements in technology and reductions in costs, PPCD/PO3G-PU elastomers could show great potential for developing high-performance tires and contributing to the sustainable growth of the tire industry.

## 2. Materials and Methods

### 2.1. Materials

PPCD (*M_n_* = 2000 g·mol^−1^) was purchased from Guangdong Dazhi Environmental Protection Technology Co., Ltd., Guangzhou, China. PO3G (*M_n_* = 2000 g∙mol^−1^) was obtained from SK Chemicals Co., Ltd. (Seongnam-si, Republic of Korea). PPDI and TME were supplied by Acros Organics, China. Additives, sulfur, and other materials were obtained as industrial-grade chemical products sold in the rubber industry. All materials were used without further processing.

### 2.2. Fabrication of PPCD/PO3G-PU Elastomers

**Scheme 1:**
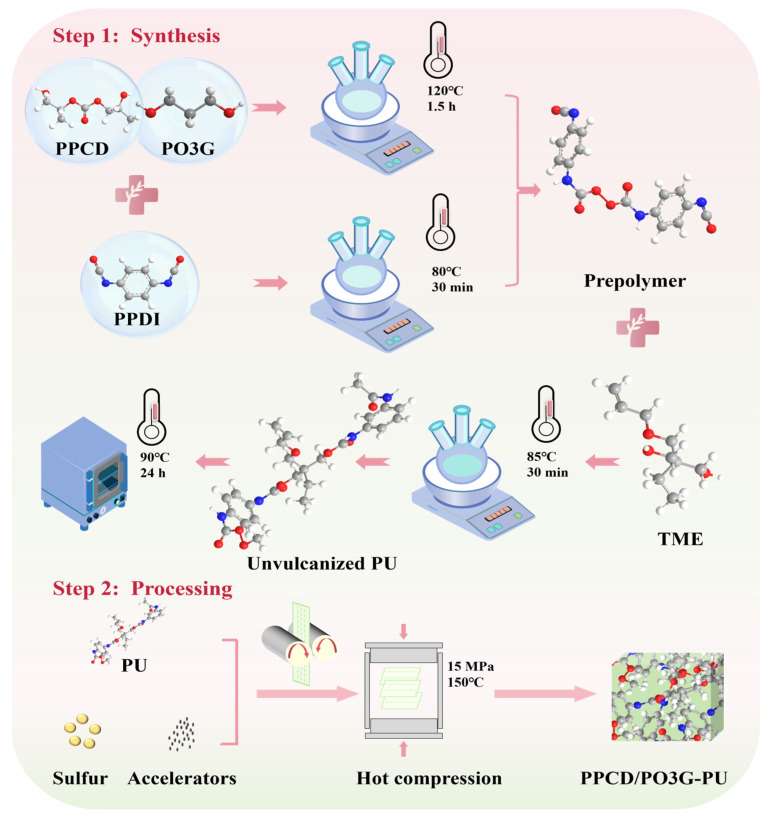
The synthesis and processing routes of PPCD/PO3G-PU elastomers.

The novel PPCD/PO3G-PU elastomers were synthesized with varying PO3G/PPCD ratios, designated as PPCD-3GX (where X denotes the percentage of PO3G, i.e., 0, 20%, 40%, 60%, 80%, and 100%, respectively). The detailed synthesis and processing route is depicted in Scheme 1. The specific synthesis procedure involved proportionally combining PPCD and PO3G in a three-necked flask, followed by stirring the mixture under vacuum for 1.5 h to remove moisture from the SS at 120 °C. Subsequently, the temperature was dropped to 80 °C and the vacuum was stopped, and N_2_ was also introduced. Then, PPDI was added and reacted for 30 min to obtain the prepolymer. Subsequently, TME was added to the prepolymer and reacted for another 30 min, and then the mixture was post-cured in an oven at 90 °C for 24 h to obtain unvulcanized elastomers. The unvulcanized elastomers were then compounded with various additives (including stearic acid, accelerators, activators, and sulfur) in an open mill to obtain vulcanized elastomers. The detailed formula is displayed in Table 1. Finally, the vulcanized elastomers were sheeted using a flat-plate vulcanizer at 150 °C and 15 MPa, which was followed by subsequent characterization.

### 2.3. Characterization

#### 2.3.1. Fourier Transform Infrared Spectroscopy (FTIR)

The unvulcanized elastomers were analyzed using an FTIR spectrometer (Bruker INVENIO-S; Germany). The analysis was performed at room temperature in the attenuated total reflection (ATR) mode, with scanning in the range of 4000–600 cm^−1^.

#### 2.3.2. Gel Permeation Chromatography (GPC)

After washing the PPCD/PO3G-PU elastomers with N,N-dimethylacetamide, the molecular weight and distribution were analyzed using GPC (Shimadzu, Japan) at a flow rate of 1.0 mL·min^−1^.

#### 2.3.3. Differential Scanning Calorimetry (DSC)

The DSC curves of the PPCD/PO3G-PU elastomers were obtained under the following conditions: a temperature range from −80 °C to 80 °C, a N_2_ flow rate of 50 mL·min^−1^, and a heating rate of 10 °C·min^−1^.

#### 2.3.4. X-Ray Diffraction (XRD)

The crystallization behavior of the PPCD/PO3G-PU elastomers was tested using an XRD (Ultima IV; Beijing, China) with a scanning temperature range from 10 °C to 50 °C and a scanning rate of 10 °C·min^−1^.

#### 2.3.5. Thermogravimetric Analysis (TGA)

The thermal stability of the PPCD/PO3G–PU elastomers was evaluated using the STARe TGA system (Mettler–Toledo International, Switzerland). The analysis was performed under a N_2_ atmosphere with a temperature range from 25 °C to 800 °C and a heating rate of 10 °C·min^−1^.

#### 2.3.6. Mechanical Performance

The mechanical properties of the PPCD/PO3G-PU elastomers were tested using a microcomputer-controlled electronic universal testing machine (CMT4503; SANS Testing Machine Co., Shenzhen, China). The specimen dimensions (50.0 mm × 6.0 mm × 2.0 mm) and tensile rate (500 mm·min^−1^) were kept consistent for all tests. The results of the five tests for each sample were averaged to minimize variability.

#### 2.3.7. Dynamic Mechanical Analysis (DMA)

DMA tests were conducted using a dynamic mechanical analyzer (GABO EPLEXOR; Gabo Instruments, Germany). The analysis was carried out in the tensile mode. The test frequency, heating rate, temperature range, and deformation were set to 10 Hz, 3 °C·min^−1^, −100 °C to 120 °C, and 0.1%, respectively. Each test was repeated three times under identical conditions, and the samples were prepared with uniform dimensions. The test results were averaged from repeated measurements to eliminate potential minor variations.

#### 2.3.8. Akron Abrasion

An Akron abrasion test was conducted using an MZ-4061 Akron abrasion machine (Mingzhu Testing Machinery Co., Ltd., Yangzhou, China). The PU wheel rotation speed was set to 76 rpm, the applied force on the PU wheel was 26.7 N, and the inclination angle between the rubber roller’s main shaft and the grinding wheel’s main shaft was 15°. After pre-grinding, the grinding wheel was operated for 1.61 km, and the wear amount of the elastomers was measured. The test was conducted at room temperature.

#### 2.3.9. Rolling Resistance

The rolling resistance of the PPCD/PO3G-PU elastomers wheels was measured using an RSS-II rolling resistance tester (Beijing Wanhuiyifang Technology Co., Ltd., Beijing, China). The test was conducted with a load of 30 kg and a rotation speed of 400 r·min^−1^ (road speed of approximately 7.54 km·h^−1^).

#### 2.3.10. Scanning Electron Microscopy (SEM)

Following the Akron abrasion test, the worn surface of the PPCD/PO3G-PU elastomers was observed using SEM (JEM-2100 F, Hitachi, Japan).

## 3. Results and Discussion

### 3.1. Structural Analysis of the PPCD/PO3G–PU Elastomers

**Figure 1:**
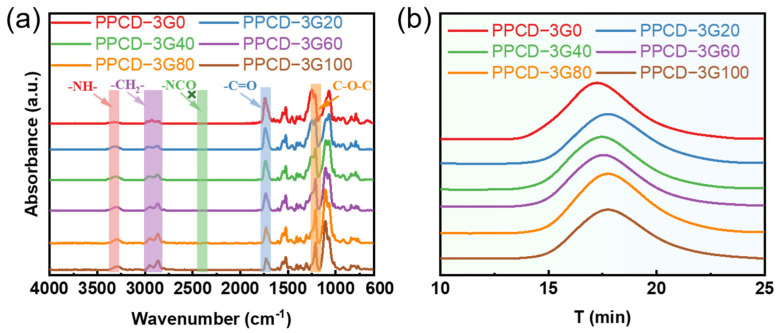
(**a**) FTIR spectra (4000 to 600 cm^−1^) and (**b**) GPC curves of PPCD/PO3G-PU elastomers.

Figure 1a shows the FTIR spectra of the PPCD/PO3G-PU elastomers in the range of 4000–600 cm^−1^. The absorption peak around 1210 cm^−1^ corresponded to the stretching vibration of ether groups, and characteristic absorption peaks of methylene groups were observed near 2950 cm^−1^ and 2865 cm^−1^ [33]. The characteristic absorption peak of the asymmetric stretching vibration of the isocyanate group typically appears at around 2270 cm^−1^, but no absorption peak was observed in this region for the elastomers, indicating complete reaction of the isocyanate groups. A stretching vibration peak of the carbonyl group was observed around 1730 cm^−1^, and that of the amino group was found at approximately 3340 cm^−1^. Figure 1b displays the GPC curves of the elastomers, with detailed data presented in Table S1. The *M_n_* value of the elastomers exceeded 6.50 × 10^4^ g·mol^−1^, and the molecular weight distribution was around 1.20. The above results confirm the successful synthesis of the PPCD/PO3G-PU elastomers [34].

### 3.2. Thermal Properties of the PPCD/PO3G-PU Elastomers

**Figure 2:**
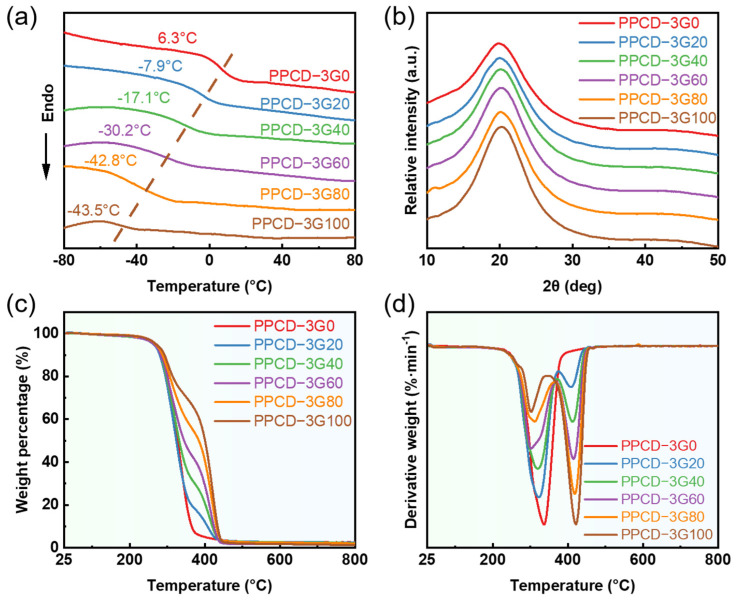
(**a**) DSC curves, (**b**) XRD curves, (**c**) TGA curves, and (**d**) DTG curves of PPCD/PO3G-PU elastomers.

It is well established that a high *T_g_* in composites makes tires more susceptible to cracking, posing risks to both safety and tire longevity. Figure 2a presents the DSC curves of the PPCD/PO3G-PU elastomers. The *T_g_* value decreased from 6.3 °C to −43.5 °C with the PO3G content rising from 0% to 100%, demonstrating that the incorporation of PO3G significantly lowered the *T_g_* value of the elastomers. The reduction in the *T_g_* value can be attributed to the presence of an odd-numbered carbon chain structure and the high concentration of ether bonds in PO3G, which imparted greater flexibility to the segments. As the proportion of PO3G increased, the number of flexible segments also rose, thereby enhancing the mobility of the molecular chains [28]. Figure 2b exhibits the XRD curves of the PPCD/PO3G-PU elastomers, where no obvious crystallization peaks were observed in all the elastomers [35], consistent with the DSC results showing no melting crystallization peaks.

Figure 2c,d express the TGA and DTG (derivative thermogravimetry) curves of the PPCD/PO3G–PU elastomers. Table S2 provides the data of the initial decomposition temperature (*T*_5%_), the temperature corresponding to the maximum weight loss rate (T_1, max_, T_2, max_), and the char residual at 800 °C of the elastomers. As the content of PO3Gincreased, the *T*_5%_ value of the elastomers remained above 401.2 °C, and the char residual at 800 °C was below 2.7%. The T_1, max_ value of the elastomers displayed a decreasing trend (from 336.1 °C to 302.1 °C), whereas the T_2, max_ value exhibited an increasing trend (from 407.6 °C to 421.8 °C), indicating good thermal stability. This observed phenomenon can be explained by the presence of a significant amount of carbonate groups in PPCD, which are known to have relatively poor thermal stability and are more susceptible to decomposition at high temperatures. As the PO3G content increased, the proportion of carbonate groups was reduced, leading to an overall improvement in the thermal stability [33].

Furthermore, PU generally displays two thermal degradation stages, corresponding to the decomposition of the HS and SS. Notably, the PPCD-3G0 elastomer presented only one thermal decomposition stage owing to the semblable thermal decomposition temperatures of PPDI and PPCD, leading to the overlapping weight loss processes of the two phases [36]. In contrast, the poor compatibility and significantly different thermal decomposition temperatures of PO3G and PPDI resulted in two distinct weight loss stages in the TGA curves [28], where the first stage corresponds to the weight loss of PPCD and PPDI, while the second stage corresponds to the weight loss of PO3G. Other researchers also found similar results [33].

### 3.3. Mechanical Properties of the PPCD/PO3G-PU Elastomers

**Figure 3:**
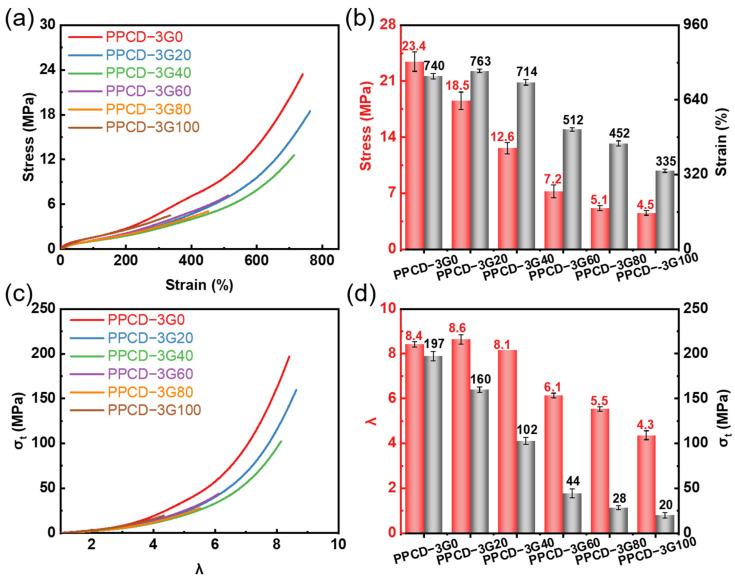
(**a**) Stress–strain curves, (**b**) stress and strain variations, (**c**) true stress (σ_t_ vs. λ) curves, and (**d**) variations in σ_t_ and λ of PPCD/PO3G-PU elastomers.

Stress–strain analysis offers valuable insights into the deformation behavior and mechanical properties of materials under practical conditions, and these are directly linked to key tire performance characteristics. A lower tensile strength can enhance tire comfort and adaptability by improving shock absorption and deformation under stress. Figure 3a,b illustrate the stress–strain relationship of the PPCD/PO3G-PU elastomers, with detailed data presented in Table S3. As the PO3G content increased, both the tensile strength (decreasing from 23.4 MPa to 4.5 MPa) and the elongation at break (decreasing from 740% to 335%) of the elastomers exhibited downward trends. The 100% elongation stress ranged from 1.1 MPa to 1.6 MPa, while the Shore A hardness remained around 60 due to the addition of the PO3G. These results could be attributed to the fact that PPCD contains a substantial number of highly polar carbonate groups with high cohesive energy and strong intermolecular forces, forming hydrogen bonds with the urethane of the elastomers. Additionally, the mechanical properties of the elastomers were further evaluated using the true stress (σ_t_) [18], which is defined as the ratio of the external load to the initial cross-sectional area of the elastomers. The σ_t_ value accounts for actual deformation and dimensional changes, providing a more accurate description of the behavior under load. Figure 3c,d exhibit that the variation in σ_t_ aligned with the stress–strain variation in the PPCD/PO3G-PU elastomers, further demonstrating that the reduction in the PPCD content led to a decrease in the mechanical properties of the elastomers.

### 3.4. Dynamic Mechanical Properties of the PPCD/PO3G-PU Elastomers

**Figure 4:**
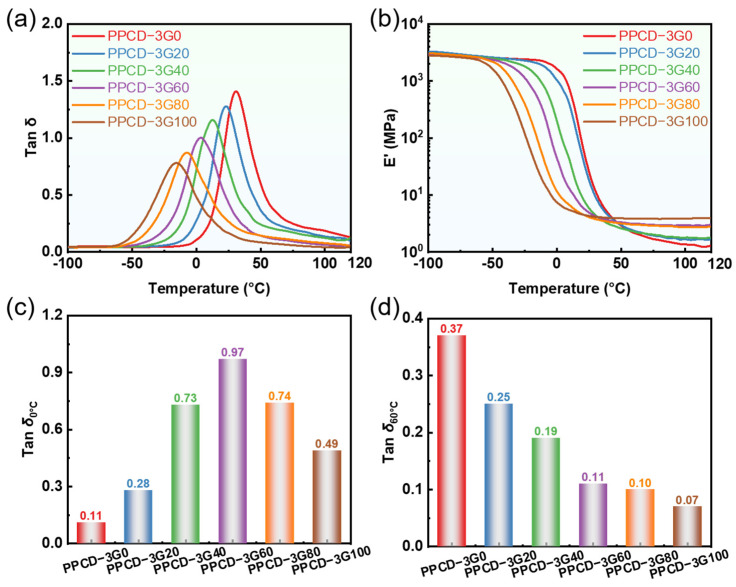
Graphs showing (**a**) tan *δ*–temperature, (**b**) E′–temperature, (**c**) tan *δ*_0°C_, and (**d**) tan *δ*_60°C_ of PPCD/PO3G-PU elastomers.

The variation in the loss factor (tan *δ*) and storage modulus (E’) of the PPCD/PO3G-PU elastomers with different PO3G contents is shown in Figure 4a,b as well as in Table S4. The results demonstrate that as PO3G content increased from 0% to 100%, the maximum loss factor (tan *δ_max_*) of the elastomers decreased from 1.41 to 0.78, and the *T_g_* value of the elastomers decreased from 30.3 °C to −15.8 °C, consistent with the DSC results. The decrease in tan *δ_max_* with the decreasing PPCD content may be attributed to the reduction in polar groups (including side methyl and carbonate groups), reducing intramolecular friction and thereby diminishing the energy dissipation capacity. Additionally, the molecular chain flexibility and mobility were enhanced with the addition of the PO3G, which facilitated the alignment of the segments and improved the regularity of their arrangement, resulting in a higher E′ and a lower *T_g_*.

In tire studies, values of tan *δ* at 60 °C and 0 °C (tan *δ*_60 °C_ and tan *δ*_0 °C_) are commonly applied to represent the rolling resistance and wet-skid resistance of tires, respectively [37,38]. A lower value of tan *δ*_60 °C_ is desired for reducing rolling resistance, while a higher value of tan *δ*_0 °C_ is preferred for enhancing wet-skid resistance. Figure 4c,d and Table S4 present the tan *δ*_60 °C_ and tan *δ*_0 °C_ values of the PPCD/PO3G-PU elastomers. As the PO3G content increased, the tan *δ*_60 °C_ value decreased to 0.07 (PPCD-3G100 elastomer), while the tan *δ*_0 °C_ value increased to 0.97 (PPCD-3G60 elastomer). The reduction in the tan *δ*_60 °C_ value could be associated with the shift in *T_g_* toward lower temperatures as the PO3G content increased. Specifically, the PPCD-3G0 elastomer was situated at the terminus of the glass transition region at 60 °C, exhibiting diminished molecular chain mobility and greater internal friction between chains compared to the rubbery state, resulting in a relatively inferior energy dissipation capacity. As the PO3G content rose, the molecular chains progressively transitioned toward the rubbery state, thereby enhancing their mobility and leading to a declining trend in the value of tan *δ*_60 °C_. The above results illustrate that the addition of PO3G enhanced the wet-skid resistance while reducing the rolling resistance of the PPCD/PO3G-PU elastomers, providing a new approach for the development of high-performance tires.

### 3.5. Rolling Resistance Properties of the PPCD/PO3G-PU Elastomers

**Figure 5:**
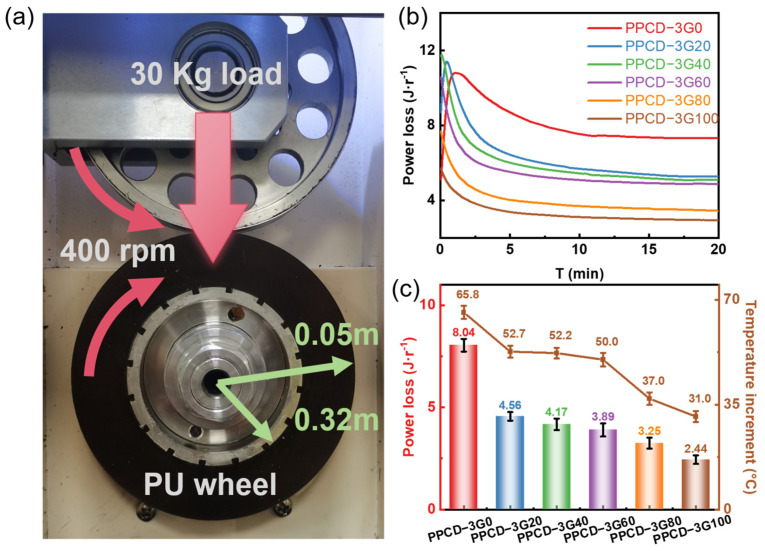
(**a**) Diagrammatic illustration of RSS-II model for rolling resistance, (**b**) energy loss curve, and (**c**) energy loss (bars) and temperature increment (solid line) of PPCD/PO3G-PU elastomers.

**Figure 6:**
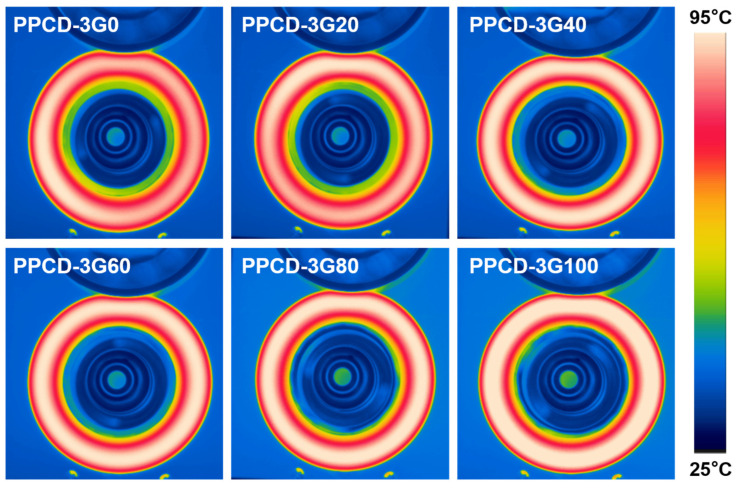
Infrared thermal images in rolling resistance test of PPCD/PO3G-PU elastomers.

A rolling resistance tester was employed to directly measure the rolling resistance of the PPCD/PO3G-PU elastomers and validate the predictive results of tan *δ*_60 °C_. Figure 5a shows the setup of the rolling resistance tester, applying a load of 30 kg to the wheel and simulating road travel at a speed of 400 rpm. Figure 5b,c display the energy loss process and temperature rise of the elastomers. With the increase in the PO3G content, the temperature rise of the elastomers decreased from 65.8 °C to 31.0 °C, and the energy loss decreased from 8.04 J·r^−1^ to 2.44 J·r^−1^, demonstrating that the rolling resistance was gradually reduced, which is consistent with the DMA results. Additionally, to more clearly and intuitively reflect the temperature changes on the elastomers’ surface, infrared thermal imaging was utilized during the testing process, where redder colors indicate higher temperature rises [39]. Figure 6 exhibits the infrared thermal images of the PPCD/PO3G-PU elastomers. It can be observed that PPCD-3G0 elastomer expressed the reddest color, while the PPCD-3G100 elastomer presented the lightest color, proving a significant decrease in the temperature rise with the increase in the PO3G content.

### 3.6. Wear Resistance Properties of the PPCD/PO3G-PU Elastomers

**Figure 7:**
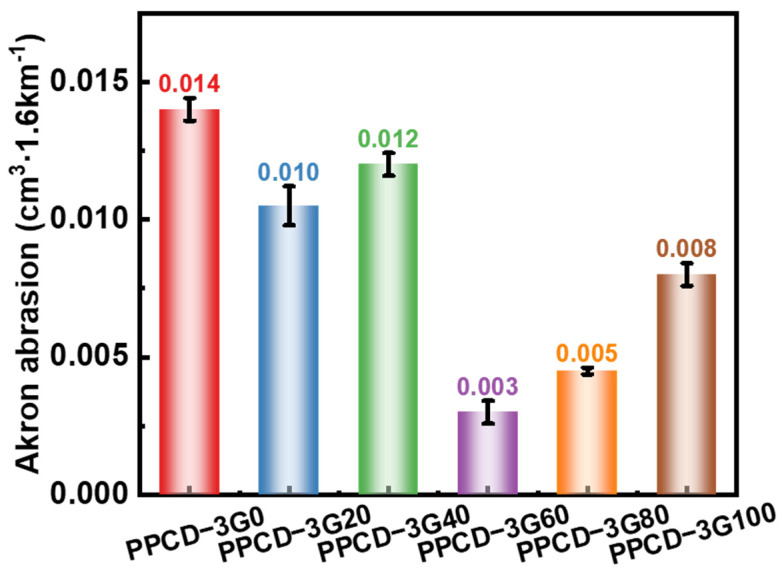
Akron abrasion of PPCD/PO3G-PU elastomers.

**Figure 8:**
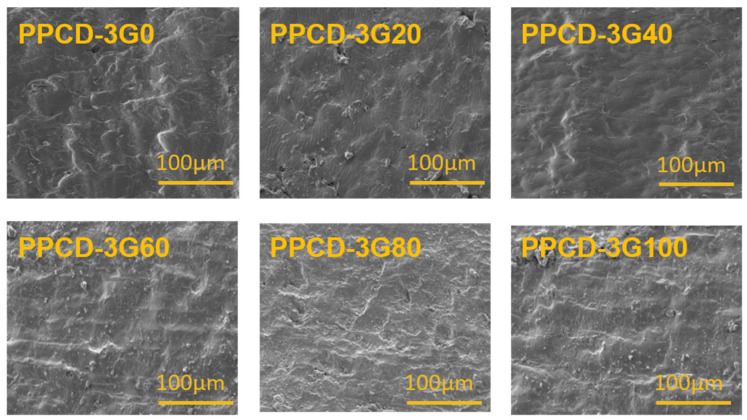
SEM images of the Akron abrasion surface topography.

High-performance tires require excellent wear resistance [40]. Akron abrasion can measure the durability of elastomer surfaces. Figure 7 presents the Akron abrasion data analysis of the PPCD/PO3G-PU elastomers. The Akron abrasion wear volumes of the elastomers were all below 0.014 cm^3^. These results demonstrate the remarkable wear resistance of the elastomers, primarily owing to the plentiful polar functional groups within PPCD. The PPCD/PO3G-PU elastomers exhibited elevated molecular cohesion energy and modulus values, thereby facilitating diminished deformation and decelerated crack propagation. Moreover, the elastomers generally exhibited Schallamach stripes on their surface after wear, and smaller spacing between these stripes indicates better wear resistance [41]. Figure 8 exhibits the Akron surface morphology of the PPCD/PO3G-PU elastomers. When the PO3G content was 0%, 20%, 40%, and 100%, Schallamach stripes could be obviously observed, and the Schallamach stripes with less wear are more densely packed. Nevertheless, the PPCD-3G60 and PPCD-3G80 elastomers did not express visible Schallamach stripes on the worn surface due to minimal wear, which further validates the Akron wear results.

**Figure 9:**
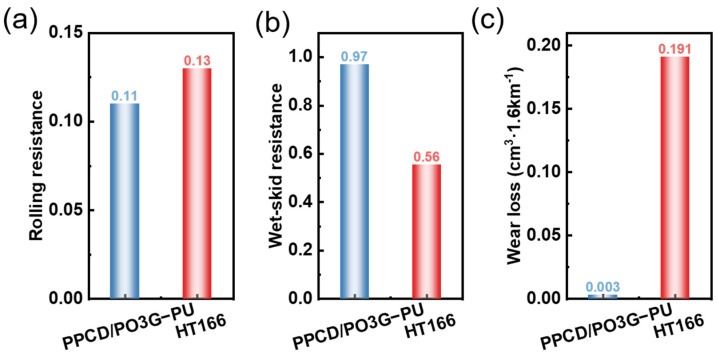
Comparison of (**a**) rolling resistance, (**b**) wet-skid resistance, and (**c**) wear loss between PPCD/PO3G-PU elastomers and HT1166.

To comprehensively evaluate the performance of the PPCD/PO3G-PU elastomers, a “magic triangle” performance comparison was conducted between the PPCD/PO3G-PU elastomers and commercially available high-performance tire elastomers, as shown in Figure 9. HT166 is a commercially available “green tire” elastomer from Linglong Co., Ltd. [42]. Compared to HT166, the PPCD/PO3G-PU elastomer displayed a 15.38% reduced in rolling resistance, a 73.21% improvement in wet-skid resistance, and a 92.11% reduction in wear. The above results illustrate that the PPCD/PO3G-PU elastomers offer significant advantages in terms of balancing the “magic triangle” by maintain low rolling resistance, high wet-skid resistance, and high wear resistance.

## 4. Conclusions

In this work, a series of PPCD/PO3G-PU elastomers were synthesized utilizing PO3G and PPCD as the SS combined with PPDI and TME as the HS. The results display that with the increase in the PO3G content, the *T_g_* value of the PPCD/PO3G-PU elastomers decreased from 6.3 °C to −43.5 °C, and the *T_5%_* value of the elastomers remained above 401.2 °C, indicating good thermal stability. In terms of tire-related characterization, the tan *δ*_60 °C_ value of the elastomers decreased to 0.07, while the tan *δ*_0 °C_ value increased to 0.97, the Akron wear volume of the PPCD/PO3G-PU elastomers remained below 0.014 cm^3^, the temperature rise decreased from 65.8 °C to 31.0 °C, and the energy loss decreased from 8.04 J·r^−1^ to 2.44 J·r^−1^, illustrating that the PPCD/PO3G-PU elastomers express low rolling resistance, high wet-skid resistance, and good wear resistance. The PPCD/PO3G-PU elastomers can reduce the reliance on petrochemical resources and effectively balance the “magic triangle” challenge in the tire industry. However, the industrial production of PPCD/PO3G-PU elastomers faces significant challenges due to cost limitations. With advancements in technology and reductions in cost, PPCD/PO3G-PU elastomers could show great potential for developing high-performance tires and contributing to the sustainable growth of the tire industry.

## Figures and Tables

**Table 1:** Vulcanized formula of PPCD/PO3G-PU elastomers.

Name	Content
Unvulcanized elastomer (phr)	100
Stearic acid (phr)	0.5
1,3-diphenylguanidine (phr)	1
Dibenzothiazole disulfide (phr)	2
Sulfur (phr)	1
NH-2 (phr)	1

* 1 phr = 1 g; NH-2 is a complex of cadmium chloride/zinc chloride and dibenzothiazole disulfide.

## Data Availability

The original contributions presented in this study are included in the article and Supplementary Material. Further inquiries can be directed to the corresponding authors.

## Supplementary Materials

The following supporting information can be downloaded at: https://www.mdpi.com/article/10.3390/ma18030603/s1, Table S1: GPC data of PPCD/PO3G-PU elastomers; Table S2: TGA and DTG data of PPCD/PO3G-PU elastomers; Table S3: Mechanical property data of PPCD/PO3G-PU elastomers; Table S4: DMA data of PPCD/PO3G-PU elastomers.
