# Supplementary material for: An Innovative Approach of Using a Bio-Based Polyurethane Elastomer to Overcome the “Magic Triangle” in Tires
Source: Materials (Basel). 2025 Jan 28;18(3):603. doi: 10.3390/ma18030603 (PMC11818608; doi:10.3390/ma18030603)
Supplement: Supplementary file 1 [file materials-18-00603-s001.zip › materials-3393552-supplementary.pdf]

Supplementary Information

An innovative approach of bio-based polyurethane elastomer to overcome the "magic triangle" in tires

Xin Wang[1,2], *Dexian Yin*[1,2], *Zhi Chen*[1,2], *Xiuying Zhao*[1,2], *Xin Ye*[1,2*], *Shikai Hu*[1,2*]

1 State Key Laboratory of Organic-Inorganic Composites, Beijing University of Chemical Technology, Beijing, 100029, China

2 Beijing Engineering Research Center of Advanced Elastomers, Beijing University of Chemical Technology, Beijing, 100029, China

This file contains **Supporting Tables S1-S4**:

Table S1: GPC data of PPCD/PO3G-PU elastomers.

Table S2: TGA and DTG data of PPCD/PO3G-PU elastomers.

Table S3: Mechanical property data of PPCD/PO3G-PU elastomers.

Table S4: DMA data of PPCD/PO3G-PU elastomers.

[*]Corresponding author: yexin@buct.edu.cn (Xin Ye)

[*]Corresponding author: skhu@mail.buct.edu.cn (Shikai Hu)

Table S1 GPC data of PPCD/PO3G-PU elastomers.

Name	M_n (10^4 g·mol^{-1})	M_w (10^4 g·mol^{-1})	$Đ$
PPCD-3G0	8.90	10.59	1.19
PPCD-3G20	7.39	8.94	1.21
PPCD-3G40	8.04	9.89	1.23
PPCD-3G60	7.54	8.90	1.18
PPCD-3G80	6.90	8.42	1.22
PPCD-3G100	6.50	8.13	1.25

Table S2 TGA and DTG data of PPCD/PO3G-PU elastomers.

Name	$T_{5\%}$ (°C)	$T_{1,\,max}$ (°C)	$T_{2,\,max}$ (°C)	Char residue at 800°C (%)
PPCD-3G0	401.2	336.1	-	2.7
PPCD-3G20	425.2	322.1	407.6	2.5
PPCD-3G40	431.7	319.9	413.4	2.3
PPCD-3G60	432.2	302.7	414.1	1.7
PPCD-3G80	438.7	312.5	418.0	1.6
PPCD-3G100	440.3	302.1	421.8	1.3

Table S3 Mechanical property data of PPCD/PO3G-PU elastomers.

Name	Tensile strength (MPa)	Elongation at break (%)	100% elongation stress (MPa)	Hardness (Shore A)
PPCD-3G0	23.4±1.2	740±11	1.6±0.2	73±3
PPCD-3G20	18.5±1.1	763±8	1.2±0.1	62±1
PPCD-3G40	12.6±0.7	714±12	1.1±0.1	59±1
PPCD-3G60	7.2±0.8	512±7	1.2±0.1	58±2
PPCD-3G80	5.1±0.3	452±11	1.2±0.2	58±1
PPCD-3G100	4.5±0.3	335±7	1.6±0.2	57±1

Table S4 DMA data of PPCD/PO3G-PU elastomers.

Name	T_g (°C)	tan δ_{max}	tan $\delta_{0°C}$	tan $\delta_{60°C}$
PPCD-3G0	30.3±0.2	1.41±0.07	0.11±0.01	0.37±0.02
PPCD-3G20	23.1±0.3	1.28±0.04	0.28±0.01	0.25±0.01
PPCD-3G40	12.4±0.2	1.16±0.06	0.73±0.05	0.19±0.01
PPCD-3G60	3.4±0.3	1.00±0.01	0.97±0.04	0.11±0.01
PPCD-3G80	-7.5±0.1	0.87±0.07	0.74±0.06	0.10±0.01
PPCD-3G100	-15.8±0.4	0.78±0.01	0.49±0.04	0.07±0.00
